# Increasing prevalence of resistance to second-line drugs among multidrug-resistant *Mycobacterium tuberculosis* isolates in Kuwait

**DOI:** 10.1038/s41598-021-87516-0

**Published:** 2021-04-08

**Authors:** Noura M. Al-Mutairi, Suhail Ahmad, Eiman Mokaddas

**Affiliations:** 1 Department of Microbiology, Faculty of Medicine, Health Sciences Centre, Kuwait University, P. O. Box 24923, 13110 Safat, Kuwait; 2 Kuwait National TB Control Laboratory, Shuwaikh, Kuwait

**Keywords:** Microbiology, Molecular biology

## Abstract

Molecular methods detect genetic mutations associated with drug resistance. This study detected resistance-conferring mutations in *gyrA*/*gyrB* for fluoroquinolones and *rrs*/*eis* genes for second-line injectable drugs (SLIDs) among multidrug-resistant *Mycobacterium tuberculosis* (MDR-TB) isolates in Kuwait. Fifty pansusceptible *M. tuberculosis* and 102 MDR-TB strains were tested. Phenotypic susceptibility testing was performed by MGIT 960 system using SIRE drug kit. GenoType MTBDR*sl* version 1 (gMTBDR*sl*v1) and GenoType MTBDR*sl* version 2 (gMTBDR*sl*v2) tests were used for mutation detection. Results were validated by PCR-sequencing of respective genes. Fingerprinting was performed by spoligotyping. No mutations were detected in pansusceptible isolates. gMTBDR*sl*v1 detected *gyrA* mutations in 12 and *rrs* mutations in 8 MDR-TB isolates. gMTBDR*sl*2 additionally detected *gyrB* mutations in 2 and *eis* mutation in 1 isolate. Mutations in both *gyrA/gyrB* and *rrs/eis* were not detected. gMTBDR*sl*v1 also detected ethambutol resistance-conferring *embB* mutations in 59 isolates. Although XDR-TB was not detected, frequency of resistance-conferring mutations for fluoroquinolones or SLIDs was significantly higher among isolates collected during 2013–2019 versus 2006–2012. Application of both tests is warranted for proper management of MDR-TB patients in Kuwait as gMTBDR*sl*v2 detected resistance to fluoroquinolones and/or SLIDs in 3 additional isolates while gMTBDR*sl*v1 additionally detected resistance to ethambutol in 58% of MDR-TB isolates.

## Introduction

Although the incidence of active disease and deaths have slowly declined worldwide in recent years, tuberculosis (TB) is still the leading cause of death from a single infectious agent^1^. According to the World Health Organization (WHO), an estimated 10 million active TB disease cases (equivalent to 130 cases per 100,000 population) and ~ 1.41 million deaths occurred in 2019^1^. Furthermore, an estimated 465,000 people developed TB that was resistant to rifampin (RR-TB), and of these, nearly 363,000 (78%) were multidrug-resistant (MDR)-TB (defined as infection with *Mycobacterium tuberculosis* strain resistant at least to rifampin, RIF and isoniazid, INH; the two most effective first-line drugs) cases^1^. The WHO has further categorized infection with *M. tuberculosis* strains resistant only to RIF and INH without additional resistance to other first-line (ethambutol, EMB and pyrazinamide, PZA) drugs as uncomplicated MDR-TB. Successful treatment of uncomplicated MDR-TB is higher compared to treatment of MDR-TB resistant to additional first-line drugs^2–5^. It is estimated that nearly 6.2% of all MDR-TB cases have XDR-TB [MDR-TB strains additionally resistant to a fluoroquinolone (FQ) plus a second-line injectable drug (SLID); kanamycin (KAN), amikacin (AMI) or capreomycin (CAP)] and at least 1 case of XDR-TB has been reported by 131 countries/territories by the end of 2018^6^. Furthermore, 20.1% of 465,000 RR/MDR-TB isolates worldwide were resistant to a FQ in 2019^1^. Worldwide, treatment success rates for drug-susceptible TB, MDR-TB and XDR-TB have been reported as 85%, 56%, and 39%, respectively^6^. Among MDR-TB strains, mutations in *rpoB* mostly confer resistance to RIF while mutations in *katG* codon 315 (*katG*), and *inhA* regulatory region (*inhA*) usually confer resistance to INH^3^. Early detection of *M. tuberculosis* in clinical specimens, its susceptibility to anti-TB drugs, and effective treatment are essential for global TB control efforts^3–7^.

The FQs, and SLIDs (KAN, AMI or CAP) are the backbone of most treatment regimens for MDR-TB as resistance to both these drugs in MDR-TB strains defines XDR-TB^2–4,8^. Patients infected with *M. tuberculosis* isolates carrying specific *gyrA* (A90V, D94G, D94N and D94Y) mutations conferring high-level resistance to FQs or *rrs* (A1401G) mutation conferring high-level resistance to KAN have higher mortality rates^9,10^. New generation FQs (Levofloxacin, LFX, moxifloxacin, MFX and gatifloxacin, GFX) have both bactericidal and sterilizing activity against *M. tuberculosis* as they penetrate into cavitary lesions and are active against resident bacterial populations^11^. With the availability of two new anti-TB drugs, bedaquiline and delamanid, FQs have recently been placed ahead of SLIDs since their use is associated with a favorable outcome for MDR-TB treatment^12^.

Phenotypic drug susceptibility testing (DST) by automated methods such as mycobacteria growth indicator tube (MGIT) 960 system, considered reliable to determine susceptibility or resistance of *M. tuberculosis* to first-line anti-TB drugs until recently, has yielded discrepant results in recent studies^3,4,7,8,10,13,14^. Furthermore, these methods are cumbersome and time-consuming, yielding results in weeks rather than few days^3,7^. Although MGIT 960 system has also been evaluated for FQs and SLIDs, these studies have mainly used *M. tuberculosis* strains exhibiting high-level resistance to these drugs^3,7,8^. Extensive data on rifampin-resistant^15–19^ and ethambutol-resistant^20–24^ strains indicate that *M. tuberculosis* isolates with low-level resistance to FQs/SLIDs may also yield discordant results by MGIT 960 system. Indeed, recent studies have shown that *M. tuberculosis* isolates with minimal inhibitory concentration (MIC) values close to the critical drug concentration (CC) and carrying specific mutations in resistance conferring (*gyrA* or *gyrB*) genes yield discordant results by phenotypic methods^25–27^. Since treatment failure or disease relapse is nearly same for RIF-resistant isolates with ‘disputed’ (MICs close to CC) or ‘canonical’ (MICs well above CC) *rpoB* mutations^28,29^, similar results are also expected for *M. tuberculosis* isolates with low-level resistance to FQs/SLIDs. The problems associated with slow and/or inaccurate DST of *M. tuberculosis* by phenotypic methods have been overcome by developing molecular methods. Although molecular methods are also not perfect^30^, specific detection of mutations for most anti-TB drugs including FQs and SLIDs correlate well with treatment outcome^8–10,15,16,28,29^.

The WHO has recommended the use of line probe assays for rapid first-line and second-line diagnostic screening for MDR-TB and XDR-TB, respectively, as the initial test in place of phenotypic DST^1,6^. This study compared the performance of GenoType MTBDR*sl* version 1 (gMTBDR*sl*v1) (containing probes targeting *gyrA* for FQ resistance, *rrs* for resistance to SLIDs and *embB* codon 306 for EMB resistance) and GenoType MTBDR*sl* version 2 (gMTBDR*sl*v2) (containing probes targeting *gyrA* and *gyrB* for FQ resistance, *rrs* for resistance to SLIDs and *eis* for resistance to KAN) tests for the detection of second-line drug resistance among MDR-TB isolates in Kuwait. Results for selected isolates were validated by PCR-sequencing of respective gene loci. Resistance to RIF and INH was also confirmed by using GenoType MTBDR*plus* or PCR-sequencing tests.

## Results

### Clinical specimens and *M. tuberculosis* isolates

The clinical source of 152 M*. tuberculosis* strains used in this study are shown in Table 1. The MDR-TB strains (n = 102, representing all available MDR-TB strains collected during 2006 to 2019) were cultured from 78 respiratory and 24 non-respiratory specimens obtained from 102 (males, n = 54) patients. Simultaneously, 50 pansusceptible *M. tuberculosis* isolates cultured from 34 respiratory and 16 non-respiratory samples collected from 50 patients (males, n = 34) were also used (Table 1). The MDR-TB strains were grown from 14 patients from Gulf Cooperation Council (GCC) countries (including 12 Kuwaiti and 2 Saudi Arabian nationals) and 88 patients from non-GCC countries (expatriate workers or their family dependents comprising Indian, n = 37; Filipino, n = 17; Ethiopian, n = 15; Nepalese, n = 6; Iraqi, n = 4; Egyptian, n = 3; Bangladeshi, n = 2; Indonesian, n = 2; Georgian, n = 1 and Syrian, n = 1 subjects). Pansusceptible strains were grown from Indian (n = 18), Filipino (n = 7), Kuwaiti (n = 6), Bangladeshi (n = 4), Nepali (n = 3), Sri Lankan (n = 3), Ethiopian (n = 2), Pakistani (n = 2), Syrian (n = 2), and 3 other (1 each from Indonesia, Sudan and Mali) patients. All isolates were cultured from newly-diagnosed TB patients before initiation of treatment with anti-TB drugs. All MDR-TB (n = 102) and pansusceptible (n = 50) isolates were identified as *M. tuberculosis* complex strains by the AccuProbe DNA probe assay^31^ and by an in-house multiplex PCR assay^32^, as expected.

**Table 1:** Clinical source of pansusceptible M. tuberculosis and MDR-TB strains.

Clinical specimens	No. of *M. tuberculosis* isolates grown as	
	Pansusceptible strains	MDR-TB strains
**Respiratory samples**		
Sputum	29	69
Bronchoalveolar lavage	5	9
**Non-respiratory samples**		
Fine needle aspirate	7	11
Pus	4	5
Lymph node	1	3
Tissue biopsy	1	3
Cerebrospinal fluid	1	2
Pleural fluid	2	0
**Total**	50	102

### Phenotypic DST patterns

The drug-susceptible *M. tuberculosis* isolates (n = 50) were susceptible to all 4 SIRE (streptomycin or STR, INH, RIF and EMB) drugs tested (pansusceptible strains). When MDR-TB strains were tested, 32 isolates were resistant to 2 (INH + RIF) drugs, 36 isolates were resistant to 3 (INH + RIF + STR = 31 and INH + RIF + EMB = 5) drugs and 34 isolates were resistant to all 4 SIRE drugs (Table 2).

**Table 2:** Phenotypic resistance by MGIT 960 system to SIRE drugs and genotypic screening of mutations in *rpoB*, *gyrA, gyrB, rrs* and *eis* among 152 clinical *M. tuberculosis* isolates.

Phenotypic resistance	No. of isolates with *rpoB* mutation as		No. of isolates detected with a mutation by gMTBDR*sl* v1/v2 in				
of *M. tuberculosis* to	S450L	Other mutations^a^	*gyrA*	*gyrB*	*rrs*	*eis*	*embB*
None (n = 50)	0	0	0	0	0	0	0
INH + RIF (n = 32)	21	11	2	0	0	0	14
INH + RIF + EMB (n = 5)	2	3	1	0	0	0	4
INH + RIF + STR (n = 31)	24	7	6^b^	0	5	0	20
INH + RIF + EMB + STR (n = 34)	26	8	3^c^	2	3^d^	1	21
Total	73	29	12	2	8	1	59

*INH* isoniazid, *RIF* rifampin, *EMB* ethambutol, *STR* streptomycin.

^a^Other mutations included H445Y/D/R, n = 12; D435V, n = 4; Q432E/K/L/P, n = 5; S450W, n = 3; V170F, n = 2; M434I + D435Y, n = 2 and D435G + H445Q, n = 1.

^b^Two isolates with *rpoB* mutation S450W.

^c^One isolate with *rpoB* mutations D435G + H445Q.

^d^One isolate with *rpoB* mutation H445R.

### Genotypic detection of resistance to RIF and INH

The GenoType MTBDR*plus* assay/PCR-sequencing data showed that all pansusceptible strains (n = 50) were susceptible to RIF (Table 2) and INH as they yielded wild-type patterns for *rpoB, katG* and *inhA*. All MDR-TB strains were resistant to RIF based on GenoType MTBDR*plus* assay/PCR-sequencing data as they exhibited an *rpoB* mutation. Of 102 MDR-TB strains, 73 and 3 isolates contained S450L and S450W mutation, respectively, while 26 isolates contained mutations at other *rpoB* codon positions (Table 2). However, GenoType MTBDR*plus* assay/PCR-sequencing data confirmed the MDR-TB status of only 100 isolates as 2 isolates exhibited wild-type pattern for *katG* and *inhA* suggesting that INH resistance in these 2 isolates either involved mutations in other regions of *katG* or *inhA* genes or in other genes^3^.

### Genotypic detection of resistance to FQs and SLIDs

All 50 pansusceptible *M. tuberculosis* isolates contained wild-type pattern for *gyrA*, *rrs* and *embB* codon 306 by gMTBDR*sl*v1 assay and for *gyrA*, *gyrB*, *rrs* and *eis* genes by gMTBDR*sl*v2 assay (Table 2). When 102 MDR-TB strains were tested by gMTBDR*sl*v1 and gMTBDR*sl*v2 assays, 12 isolates contained a mutation in *gyrA* (A90V,3; D94G, n = 5; D94A, n = 2 and D94N/Y, n = 2) while the remaining 90 isolates contained wild-type *gyrA*. Of 2 isolates with D94N/Y mutations in *gyrA*, PCR-sequencing identified D94N and D94Y mutation in 1 isolate each. Of 12 isolates with *gyrA* mutations, 2 isolates were resistant to INH + RIF only, 7 isolates were resistant to 3 while the remaining 3 isolates were resistant to all 4 SIRE drugs (Table 2). Furthermore, gMTBDR*sl*v1 and gMTBDR*sl*v2 assays detected 8 other isolates with A1401G mutation while the remaining 94 isolates contained wild-type *rrs*. Of 8 isolates with an *rrs* mutation, 5 isolates were resistant to STR + INH + RIF while the remaining 3 isolates were resistant to all 4 SIRE drugs (Table 2).

The gMTBDR*sl*v1 assay also detected an *embB* codon 306 mutation in 59 MDR-TB strains (M306V, n = 32; M306I, n = 17 and 10 isolates with a non-specific mutation detected by lack of hybridization with wild-type probe) while the remaining 43 isolates contained wild-type *embB* codon 306. Among 10 isolates with a non-specific *embB* mutation, PCR-sequencing of *embB* identified M306I (ATG306ATC) mutation in 6 isolates and M306L (ATG306CTG) mutation in 4 isolates. Of 59 MDR-TB isolates with *embB* codon 306 mutations, 14 isolates were resistant to INH + RIF only, 24 isolates were resistant to 3 while the remaining 21 isolates were resistant to all 4 SIRE drugs (Table 2).

The gMTBDR*sl*v2 assay also detected N538D mutation in 1 isolate and a non-specific mutation indicated by lack of hybridization with wild-type (WT) *gyrB* probe in another isolate. PCR-sequencing of *gyrB* identified T539N mutation in the latter isolate. The remaining 100 isolates contained wild-type *gyrB*. Furthermore, 1 isolate contained an *eis* mutation detected only by lack of hybridization with WT2 *eis* probe. PCR-sequencing of *eis* identified G-10A mutation in this isolate. The remaining 101 isolates contained wild-type *eis*. Both *eis* and *gyrB* mutations were detected in isolates resistant to all 4 SIRE drugs (Table 2). The prevalence of mutations conferring resistance to FQs (*gyrA/gyrB*) or SLIDs (*rrs/eis*) was significantly higher in MDR-TB strains resistant to 3 drugs versus 2 drugs (12 of 36 versus 2 of 32, *P* = 0.007) as well as for all 4 SIRE drugs versus 2 drugs (9 of 34 versus 2 of 32, *P* = 0.045). Interestingly, the prevalence of mutations conferring resistance to FQs (*gyrA/gyrB*) or SLIDs (*rrs/eis*) was also higher in MDR-TB strains with an *rpoB* S450L mutation versus isolates with other *rpoB* mutations (19 of 73 versus 4 of 29, *P* = 0.182), however, the difference was not statistically significant.

The distribution of mutations in *gyrA/gyrB* or *rrs/eis* genes conferring resistance to FQs and SLIDs, respectively, among 23 MDR-TB strains collected over a 14-year period (2006 to 2019) together with mutations in *rpoB*, *inhA* and *embB* genes and fingerprinting data by spoligotyping are presented in Table 3. No isolate contained a mutation in both *gyrA/gyrB* + *rrs/eis* genes, i.e., XDR-TB was not detected in Kuwait (Table 3). The spoligotyping data showed that the majority (13 of 21, 62%) of MDR-TB strains with a mutation in *gyrA/gyrB* or *rrs/eis* genes belonged to Beijing genotype. All isolates with identical *gyrA* mutation were genotypically distinct strains based on other mutation patterns and/or by spoligotyping (Table 3). However, 6 of 8 isolates with A1401G mutation in *rrs* were genetically identical. The year-wise data on the prevalence of *gyrA/gyrB* mutations conferring resistance to FQs, *rrs/eis* mutation conferring resistance to SLIDs and *embB* codon 306 mutation conferring resistance to EMB in MDR-TB strains during the 14-year study period are shown in Table 4. When the total 14-year period of the study was split into two 7-year periods, the prevalence of mutations conferring resistance to FQs (9 of 47 versus 5 of 55, *P* = 0.160) or SLIDs (7 of 47 versus 2 of 55, *P* = 0.077) alone was higher in MDR-TB strains collected during 2013 to 2019 compared to 2006 to 2012, however, the differences were not statistically significant (Table 4). On the contrary, the prevalence of mutations conferring resistance to FQs or SLIDs (pre-XDR-TB) was significantly higher during 2013 to 2019 compared to 2006 to 2012 (16 of 47 versus 7 of 55, *P* = 0.016) (Table 4).

**Table 3:** Clinical details and phenotypic and molecular characterization of 23 MDR-TB isolates by GenoType MTBDR*sl* version 1 and GenoType MTBDR*sl* version 1 showing genotypic resistance to FQs or SLIDs^a^.

Serial no	Clinical specimen^a^	Isolate no	Year of isolation	Phenotypic resistance to^b^	Drug resistance-conferring mutation(s) detected in^c^							Spoligotyping data	
					*rpoB*	*inhA*	*gyrA*	*gyrB*	*rrs*	*eis*	*embB*^*d*^	SIT^e^	Family
1	Sputum	8728/40	2006	SIRE	D435G + H445Q	WT	D94A	WT	WT	WT	WT	44	T5
2	Sputum	3370/305	2008	IRE	S450L	G-17 T	D94G	WT	WT	WT	M306V	26	CAS1-Delhi
3	CSF	8714/770	2009	SIRE	S450L	WT	WT	T539N	WT	WT	M306V	244	Beijing
4	Sputum	14,626/1189	2009	SIRE	H445R	WT	WT	WT	A1401G	WT	M306V	1	Beijing
5	Sputum	7596/666	2010	SIR	S450L	WT	D94A	WT	WT	WT	WT	49	H3
6	BAL	1185/768	2011	IR	S450L	WT	D94G	WT	WT	WT	M306I	288	CAS2
7	Pus	3762/322	2011	SIR	S450L	WT	WT	WT	A1401G	WT	WT	1	Beijing
8	LN	1266/96	2013	SIRE	S450L	WT	WT	N538D	WT	WT	WT	11	EAI3-IND
9	Sputum	3608/216	2013	SIR	S450L	WT	A90V	WT	WT	WT	M306V	1	Beijing
10	Sputum	463/57	2014	SIR	S450W	WT	WT	WT	A1401G	WT	WT	1	Beijing
11	BAL	16,684/10	2014	SIR	S450L	WT	D94N	WT	WT	WT	M306V	794	CAS1-Delhi
12	Sputum	818/153	2015	SIR	S450L	WT	WT	WT	A1401G	WT	WT	1	Beijing
13	Sputum	2723/225	2015	SIRE	S450L	WT	D94G	WT	WT	WT	M306V	1	Beijing
14	Sputum	6633/640	2015	SIRE	S450L	WT	WT	WT	A1401G	WT	WT	1	Beijing
15	Sputum	10,246/708	2015	SIR	S450L	WT	WT	WT	A1401G	WT	WT	1	Beijing
16	Sputum	4784/255	2016	SIR	S450W	WT	A90V	WT	WT	WT	M306I	NA	Orphan
17	Sputum	5089/283	2016	SIR	S450L	T-8A	WT	WT	A1401G	WT	M306V	1	Beijing
18	Sputum	5873/294	2016	SIR	S450L	WT	D94G	WT	WT	WT	WT	26	CAS1-Delhi
19	Sputum	12,373/650	2016	IR	S450L	WT	D94Y	WT	WT	WT	WT	1	Beijing
20	Sputum	7730/394	2017	SIRE	S450L	WT	WT	WT	WT	G-10A	WT	1	Beijing
21	Sputum	16,359/764	2017	SIRE	S450L	WT	WT	WT	A1401G	WT	WT	1	Beijing
22	Sputum	13,820/604	2018	SIRE	S450L	C-15 T	A90V	WT	WT	WT	M306V	ND	NA
23	CSF	14,406/637	2019	SIR	S450L	WT	D94G	WT	WT	WT	M306I	ND	NA

^a^FQs, fluoroquinolones, SLIDs, second-line injectable drugs; CSF, cerebrospinal fluid; BAL, bronchoalveolar lavage; LN, lymph node.

^b^S, streptomycin; I, isoniazid; R, rifampin, E, ethambutol.

^c^All isolates contained S315T mutation in *katG* gene.

^d^*embB* codon 306 mutations only; ^e^SIT, shared international type; NA, not available; ND, not done.

**Table 4:** The year-wise prevalence of *gyrA/gyrB, rrs/eis* and *embB* mutations in multidrug-resistant *M. tuberculosis* (MDR-TB) isolates analyzed during the 14-year study period.

Year	No. of MDR-TB	No. of MDR-TB isolates with a mutation in				
	Isolates tested	*gyrA*	*gyrB*	*rrs*	*eis*	*embB*^a^
2006	8	1	0	0	0	2
2007	10	0	0	0	0	5
2008	7	1	0	0	0	4
2009	5	0	1	1	0	4
2010	6	1	0	0	0	2
2011	10	1	0	1	0	7
2012	9	0	0	0	0	5
2013	5	1	1	0	0	4
2014	8	0	0	1	0	5
2015	10	2	0	3	0	6
2016	8	3	0	1	0	5
2017	7	0	0	1	1	2
2018	5	1	0	0	0	4
2019	4	1	0	0	0	4
Total	102	12	2	8	1	59

^a^*embB* codon 306 mutations only.

## Discussion

Kuwait is an Arabian Gulf country in the Middle East. The total population of 4.8 million individuals in 2019 comprised 30% Kuwaitis and 70% expatriate workers or their dependents (https://www.paci.gov.kw/Default.aspx). Most expatriates in Kuwait originate from TB endemic countries of South/Southeast Asia and Africa (such as India, Bangladesh, Pakistan, Philippines, Egypt, Sudan and Ethiopia)^31,33,34^. Kuwait has a low (~ 24 cases per 100 000 population) incidence of TB and a low (~ 1.1%) incidence of MDR-TB^31,35^. Nearly 85% of all TB cases and > 90% of MDR-TB cases occur in expatriate population^31,35^. These cases mainly arise due to reactivation of latent TB infection acquired previously by TB patients in their respective countries even though all expatriates are screened for the evidence of active TB disease at the time of their entry into Kuwait^33–35^. All clinical specimens from suspected TB patients are tested by GeneXpert MTB/RIF assay for rapid diagnosis of active TB disease and detection of RIF resistance in addition to routine processing for smear microscopy and culture by MGIT 960 system^30,31^. Although phenotypic DST against first-line drugs and STR is performed on all *M. tuberculosis* isolates for optimal patient management, phenotypic DST against second-line drugs is not performed, mainly due to low rate of MDR-TB^31,35^.

In this study we evaluated the performance of two line probe assays (gMTBDR*sl*v1 and gMTBDR*sl*v2) for rapid detection of mutations conferring resistance to FQs and SLIDs in *M. tuberculosis* and the results were extended by PCR-sequencing of respective gene loci. These assays identified 5 different *gyrA* mutations at 2 different codon positions (A90V, 3; D94G, n = 5; D94A, n = 2; D94N, n = 1 and D94Y, n = 1) in 12 MDR-TB isolates in Kuwait which are also the most frequently observed *gyrA* mutations from some geographical locations^26,27,36–40^. Furthermore, *M. tuberculosis* isolates carrying D94G, D94N and D94Y mutations in *gyrA* exhibit high-level resistance to FQs and cause higher mortality rates among patients infected with such strains^9,10^. Application of gMTBDR*sl*v2 and PCR-sequencing further identified *gyrB* mutations (N538D and T539N) in 2 additional isolates which have also been described previously among FQ-resistant *M. tuberculosis* isolates^36–41^.

In a systematic review based on 46 studies involving 3,846 unique *M. tuberculosis* isolates with different phenotypic resistance profiles to FQs, Avalos et al.^42^ showed that mutations G88C, S91P, D94G and D94Y in quinolone resistance determining region of *gyrA* are always associated with FQ-resistant strains. Similarly, N538D and T539N in quinolone resistance determining region of *gyrB* were also always associated with FQ-resistant strains. On the contrary, mutations A90V, D94A and D94N in *gyrA* were observed in 330 of 1995, 177 of 1995 and 122 of 1995 ofloxacin-resistant isolates but also in 4 of 1572, 1 of 1572 and 1 of 1572 ofloxacin-susceptible isolates, respectively^42^. Several studies have shown that A90V and D94A mutations cause low-level resistance (minimum inhibitory concentrations, MICs of 0.5 mg/L to 4 mg/L) to ofloxacin/levofloxacin while D94G and D94N mutations mostly cause high-level resistance to FQs (MICs of 4 mg/L to 32 mg/L)^27,37–39,43^. Phenotypic DST of *M. tuberculosis* for first-line anti-TB drugs; RIF, EMB and PZA is problematic and many isolates with low-level yet clinically significant resistance to RIF and EMB are scored as drug-susceptible strains^15–20,28,29,44^. Similarly, phenotypic DST for FQs is also not perfect as some isolates with low-level resistance and containing specific *gyrA* (mainly A90V and D94A) mutations yield discordant results^25,27,38^. Taken together, the findings suggest that detection of *gyrA* mutations in few FQ-susceptible isolates is also very likely due to faulty phenotypic DST data rather than lack of association of *gyrA* mutations with FQ resistance.

All 23 isolates with *gyrA/gyrB* or *rrs/eis* mutation contained S315T mutation in *katG* and 19 of 23 isolates contained S450L mutation in *rpoB*. The mutation S315T in *katG* has minimal effects on fitness of *M. tuberculosis* and isolates with this mutation are more likely to remain virulent, are amenable to further transmission and so acquire resistance to additional drugs^3,8,45^. Similarly, the mutation S450L in *rpoB* has minimal effects on fitness as *M. tuberculosis* isolates with this mutation usually contain compensatory mutations in *rpoA*/*rpoC*, remain infectious and thus acquire resistance to additional drugs in new patients^3,8,45,46^. Although the prevalence of mutations conferring resistance to FQs (*gyrA/gyrB*) and SLIDs (*rrs/eis*) in MDR-TB strains was higher in isolates with an *rpoB* S450L mutation versus isolates with all other mutations (19 of 73 versus 4 of 29), the difference did not reach statistical significance (*P* = 0.182). High prevalence of *gyrA* and *rrs* mutations conferring resistance to FQs and SLIDs, respectively, in MDR-TB strains carrying S450L mutation in *rpoB* gene was also reported from India^47^. The mutation patterns and fingerprinting data by spoligotyping showed that nearly all 14 isolates with a *gyrA/gyrB* mutation were genotypically distinct strains. These 14 isolates were obtained from expatriate patients with 13 of 14 patients originating from the Indian subcontinent (India, n = 11, Bangladesh, n = 1; Nepal, n = 1). The data are consistent with previous observations showing that most MDR-TB strains in Kuwait are genotypically different as they arise mostly as a result of reactivation of latent *M. tuberculosis* infection acquired previously by expatriate patients in their respective countries^34,48,49^. Furthermore, the occurrence of *gyrA* mutations in MDR-TB strains isolated mostly from Indian patients is consistent with high prevalence of resistance of multidrug-resistant *M. tuberculosis* isolates from India to FQs^40,47,50,51^. Also, the detection of D94G (n = 5) and A90V (n = 3) mutations in 8 of 10 FQ-resistant strains is consistent with the high prevalence of these mutations in MDR-TB strains from India^40,47,50,51^.

Eight MDR-TB isolates contained A1401G mutation in *rrs* that confers resistance to SLIDs and 6 of 8 isolates (isolate no. 3762/322, 463/57, 818/153, 6633/640, 10,246/708 and 16,359/764) were genetically similar and were recovered from Kuwaiti patients, likely as a result of local transmission of MDR-TB in Kuwait even though the index case remained elusive^34^. Although phenotypic DST against SLIDs was not performed in this study, all MDR-TB isolates with a defined *rrs* mutation in a recent study from South Africa showed phenotypic resistance to SLIDs^39^. A multicenter cohort study carried out in seven high TB burden countries has recently shown that inaccurate DST by phenotypic methods leads to under-treatment of drug-resistant TB and increased patient mortality^52^. The study further showed that rapid molecular DST in place of phenotypic DST for first-line and second-line drugs is required for improved outcome for patients with MDR-TB, pre-XDR-TB or XDR-TB^52^.

Although XDR-TB was not detected in Kuwait, the prevalence of mutations conferring resistance to FQs alone (9 of 47 versus 5 of 55, *P* = 0.160) or SLIDs alone (7 of 47 versus 2 of 55, *P* = 0.077) was higher, though not statistically significant, in MDR-TB strains collected during 2013 to 2019 compared to 2006 to 2012. However, the prevalence of mutations conferring resistance to FQs or SLIDs (pre-XDR-TB) was significantly higher during 2013 to 2019 compared to 2006 to 2012 (16 of 47 versus 7 of 55, *P* = 0.016) strongly suggesting that the prevalence of these mutations is increasing in Kuwait. The prevalence of resistance to FQs has also increased among MDR-TB strains in recent years in India^53–56^ and China^43^. These findings are relevant as most (11 of 14, 79%) MDR-TB strains in Kuwait with *gyrA/gyrB* mutations were obtained from expatriate patients of Indian origin.

Application of gMTBDR*sl*v1 assay also detected an *embB* codon 306 mutation in 59 of 102 (58%) MDR-TB strains. Interestingly, only 25 of these 59 isolates were phenotypically resistant to EMB while the remaining 34 isolates were EMB-susceptible. The *embB* codon 306 mutations confer low-level resistance to EMB which is often missed by the faulty phenotypic DST methods^20–22,24,44^. Thus rapid detection of EMB resistance in MDR-TB strains by gMTBDR*sl*v1 test will avoid treatment with an ineffective drug, evolution of further resistance and drug-related toxicity^57^.

Our study has a few limitations. (1) Phenotypic DST of pansusceptible and MDR-TB strains against FQs and SLIDs was not performed, (2) The presence of mutations in *gyrA/gyrB* genes conferring resistance to FQs and in *rrs/eis* genes conferring resistance to SLIDs by gMTBDR*sl*v1 and gMTBDR*sl*v12 tests was carried out in culture isolates and not directly on clinical samples obtained from TB patients and (3) The outcome of infection among MDR-TB patients was not available as most patients were expatriates who were sent back to their respective countries after the initial objective of sputum smear-negative status was achieved.

In conclusion, our results show that combined use of gMTBDR*sl*v1 and gMTBDR*sl*v2 assays in 102 MDR-TB strains detected mutations in 12 and 2 isolates in *gyrA* and *gyrB,* respectively conferring resistance to FQs and in 8 and 1 isolate in *rrs* and *eis*, respectively, conferring resistance to SLIDs. Mutations in *embB* were also detected in 59 MDR-TB isolates. These mutations were absent among 50 pansusceptible *M. tuberculosis* isolates. Although XDR-TB was not detected, an increasing trend in the frequency of pre-XDR-TB was evident in Kuwait as the frequency of mutations conferring resistance to FQs or SLIDs was significantly higher among isolates collected during 2013–2019 versus 2006–2012. Application of both tests is warranted for proper management of MDR-TB patients in Kuwait, a low TB/MDR-TB setting, as gMTBDR*sl*v2 detected resistance to FQs and/or SLIDs in 3 additional isolates while gMTBDR*sl*v1 additionally detected resistance to EMB in 58% of MDR-TB isolates. The *embB* mutations confer low-level resistance to EMB which is often missed by the faulty phenotypic DST methods and their detection in MDR-TB strains by gMTBDR*sl*v1 will avoid treatment with an ineffective drug and drug-related toxicity.

## Materials and methods

### Clinical specimens and *M. tuberculosis* isolates

A total of 102 MDR-TB isolates cultured during 2006 to 2019 from 102 TB patients (representing all available MDR-TB strains) and 50 randomly selected *M. tuberculosis* isolates fully susceptible to first-line anti-TB drugs (pansusceptible strains) collected from 50 TB patients were analyzed. The MDR-TB strains were grown from 78 respiratory and 24 non-respiratory specimens while pansusceptible strains were cultured from 34 respiratory and 16 non-respiratory clinical specimens. All clinical specimens were obtained from suspected TB patients after obtaining verbal consent only as part of routine patient care, diagnostic work-up and resistance surveillance at Kuwait National TB Control Laboratory (KNTCL) before initiation of treatment with anti-TB drugs. The study was approved by the Health Sciences Center Ethical Committee, Kuwait University (Approval no. VDR/EC/3451 dated 18-12-2018) and all experimental procedures and investigations were performed in accordance with their guidelines and regulations. Since the study did not involve direct contact with patients and the results are reported on deidentified samples without revealing patient identity, the need for informed consent was waived by the Health Sciences Center Ethical Committee.

Sterile clinical specimens were processed directly for the cultivation of mycobacteria whereas non-sterile samples were processed by using *N*-acetyl-L-cysteine and sodium hydroxide (NALC/NaOH) at KNTCL for culture. The cultures were grown in liquid media-based automated mycobacteria growth indictor tube (MGIT) 960 system according to manufacturer’s instructions (Becton Dickinson, Sparks, MD, USA) and as described previously^31,44^. All MGIT cultures were positive for the presence of acid-fast bacilli (AFB) by Ziehl–Neelsen smear microscopy and for the presence of *M. tuberculosis* complex DNA by AccuProbe DNA probe assay, performed as described previously^31^.

### Phenotypic DST by Bactec MGIT 960 system

Phenotypic DST of all *M. tuberculosis* isolates was performed with the automated MGIT 960 system for RIF, INH, EMB and STR by using the SIRE drug kit used according to manufacturer’s instructions and as described previously^31,44^. The KNTCL regularly participates in drug susceptibility proficiency testing.

### Genotypic characterization

Genotypic characterization was performed at Reference Mycobacteriology Laboratory, Department of Microbiology, Faculty of Medicine, Kuwait University. For this purpose, genomic DNA was obtained from MGIT 960 system cultures by using Chelex-100 as described previously^58^. All MGIT 960 system cultures were first tested for the presence of *M. tuberculosis* complex DNA by an in-house multiplex PCR assay, performed as described previously^32^, and for mutations in genes conferring resistance to first-line (RIF and INH) and second-line (FQs and SLIDs) anti-TB drugs.

### Detection of mutations conferring resistance to RIF and INH

All isolates were tested by commercial GenoType MTBDR*plus* line probe assay (Hain Lifesciences, Nehren, Germany) for the detection of mutations in hot-spot region of *rpoB* gene for RIF resistance and for detection of mutations at *katG* codon 315 (*katG*) and *inhA* regulatory region (*inhA*) for INH resistance, according to kit instructions and as described previously^59^. Water was used in place of DNA for negative controls. PCR-sequencing of hot-spot region and N-terminal region of *rpoB* gene and *katG* and *inhA* was carried out for isolates showing no mutation or a non-specific mutation indicated by lack of hybridization with a wild-type probe only. DNA sequencing was performed as described previously^59^. *M. tuberculosis* codon numbering system was used for the *rpoB* gene^60^.

## Detection of mutations conferring resistance to FQs and SLIDs

The gMTBDR*sl*v1 contains probes targeting *gyrA* for FQ resistance, *rrs* for resistance to SLIDs and *embB* gene codon 306 for EMB resistance. The gMTBDR*sl*v2 contains probes targeting *gyrA* and *gyrB* for FQ resistance, *rrs* for resistance to SLIDs and *eis* for resistance to KAN^36^. Both gMTBDR*sl*v1 and gMTBDR*sl*v2 were obtained commercially (Hain Lifesciences) and were used according to kit instructions. Lack of detection of specific mutation or detection of resistance only by lack of hybridization with wild-type probes was confirmed by PCR amplification followed by DNA sequencing (PCR-sequencing) of the corresponding gene fragment.

The *gyrA* gene was amplified by using GYRAF (5′-CGCAGCTACATCGACTATGCGATG-3′) and GYRAR (5′-GGGATGAAATCGATGTCTCCTCG-3′) primers and PCR amplification protocol described previously^58^, the amplicons were purified by using PCR product purification kit (Qiagen, Hilden, Germany) used according to kit instructions and both strands were sequenced by using internal sequencing primer GYRAFS (5′-CGGGTGCTCTATGCAATGTTC-3′) or GYRARS (5′-GGCTTCGGTGTACCTCATCGCC-3′) by using the DNA sequencing protocol described previously^34,59^. The *gyrB* gene was amplified by using GYRBF (5′-CAAGTCCGAACTGTATGTCGTA-3′) and GYRBR (5′-CCGCATGAACCGGAACAACAA-3′) primers, the amplicons were purified and both strands were sequenced as described above except that sequencing primer GYRBFS (5′-TCCGAACTGTATGTCGTAGAAG-3′) or GYRBRS (5′-ATGAACCGGAACAACAACGTCA-3′) was used.

The 3′-end of *rrs* (16S rRNA) gene was amplified by using 16S3F (5′-GCGATGCCGCGAGGTTAAGCGAA-3′) and 16S3R (5′-CCAACAGTGTGTTGGTGGCCAA-3′) primers, the amplicons were purified and both strands were sequenced as described above except that 16S3FS (5′-ATCCTTAAAAGCCGGTCTCAGT-3′) or 16S3RS (5′-CTCCTTAGAAAGGAGGTGATCCA-3′) was used as sequencing primer. The 5′-end of *eis* gene was amplified by using EIS5F (5′-CCAGCGTAACGTCACGGCGAA-3′) and EIS5R (5′-GCACCGTCAACCGCAGATCCA-3′) primers, the amplicons were purified and both strands were sequenced as described above except that EIS5FS (5′-CACGGCGAAATTCGTCGCTGA-3′) or EIS5RS (5′-GCAGATCCATGTACAGCGCCA-3′) was used as sequencing primer. The *embB* codon 306 region was amplified by using EMB306A (5′-CCGACGCCGTGGTGATATTCGGCT-3′) and EMB306B (5′-GTAATACCAGCCGAAGGGATCCTC-3′) primers and the purified amplicons were sequenced as described previously^20^. Nucleotide and amino acid sequences of the amplified products were compared with wild-type sequence from *M. tuberculosis* H_37_Rv using Clustal omega.

### Spoligotyping

Fingerprinting of *M. tuberculosis* isolates carrying mutations in target genes conferring resistance to FQs and SLID was carried out by spoligotyping, performed as described previously^34^. The spoligotyping results in binary format were used for the assignment of different phylogenetic lineages according to SITVIT database (http://www.pasteur-guadeloupe.fr:8081/SITVITDemo/index.jsp). The spoligotyping patterns not described previously in SITVIT2 database were designated as 'orphan' patterns^34^.

### Statistical analyses

Statistical analyses were performed by using Fisher’s exact (two-tailed) test or Pearson’s chi-square test as appropriate and probability levels < 0.05 were considered as significant. Statistical analyses were performed by using WinPepi software ver. 11.65 (PEPI for Windows, Microsoft Inc., Redmond, WA, USA).
